# A NICE approach to addressing health inequalities in breast cancer guidance

**DOI:** 10.1002/gin2.12015

**Published:** 2024-04-24

**Authors:** Eric Slade, Kirsty Luckham, Lesley Owen

**Affiliations:** ^1^ National Institute for Health and Care Excellence London UK

**Keywords:** breast cancer, clinical guidelines, health inequalities

## Abstract

- NICE seeks to reduce health inequalities through its guidance.
- NICE uses equality and health inequalities assessment (EHIA) to identify and address health inequalities.
- A health inequalities briefing was developed for breast cancer‐related guidance to consider and address health inequalities more systematically.
- The committee made recommendations that consider the impact of health inequalities on access and adherence.
- Further research is recommended for interventions targeting specific population subgroups.
- The health inequalities briefing identified late diagnosis and screening variations as key issues among deprived women and ethnic minority groups, which was passed on to the National Institute for Health and Care Research as a research priority.

## 1 INTRODUCTION TO HEALTH INEQUALITIES

Health inequalities are differences in health across the population and between different groups in society that are systematic, unfair and avoidable. They are caused by the conditions in which people are born, live, work and grow. These conditions influence peoples' opportunities for good mental and physical health.^1^

Reducing health inequalities is one of NICE's core principles. NICE's guidance supports strategies that improve population health as a whole while offering particular benefits to the most disadvantaged. Adopting NICE's recommendations into practice will ensure the care provided is effective and consistent and makes efficient use of resources. And ultimately, it reduces the impact of health inequalities on people's health.^2^

Generally, a form called Equality and Health Inequalities Assessment (EHIA) is created when developing each guidance topic. EHIA records the approaches used to identify potential equality and health inequalities issues, identifies inequalities issues and how these were considered and addressed at each stage of the guideline development process. However, the EHIA is largely based on the input from the developers and topic experts as well as the health inequalities raised by committee members. Further information on our process and methods can be found in our guidelines manual.^3^

NICE is exploring new approaches to addressing health inequalities in guidance development. One approach used, and the focus of this brief research report, is the development of the overarching health inequalities briefing to inform health inequality issues on any breast cancer‐related topic update.

## 2 THE APPROACH TO BREAST CANCER‐RELATED GUIDANCE

The health inequalities briefing for breast cancer was a pragmatic, targeted review of evidence exploring the health inequalities associated with breast cancer. It aimed to support both the NICE development team and the committee during breast cancer guidance development to consider health inequalities issues more systematically and transparently. The findings within the briefing also highlighted key gaps in evidence, potential research questions and research recommendations not only to NICE but to the wider health and care system from a health inequalities perspective.

A small technical team was formed to develop a briefing protocol, conduct searches, review and summarise research, interpret findings and undertake independent quality assurance. In the health inequalities briefing, the King's Fund framework (Table 1) for health inequalities was used to synthesise examples of the key health inequalities faced by populations in England across the four dimensions of inequality and five levels of outcomes for each dimension.

**Table 1:** The King's Fund framework for health inequalities adapted to breast cancer briefing.

Inequalities between who?		Inequalities of what?	
Protected characteristics	E.g., Age, disability, ethnicity, sex, gender reassignment and sexual orientation	Health status	E.g., Life expectancy, breast cancer incidence and prevalence, breast cancer outcomes, breast cancer mortality
Socioeconomic deprivation	E.g., Deprivation as measured using The Index of Multiple Deprivation	Behavioural risks to health	E.g., Obesity, smoking, alcohol consumption, physical activity, hormone therapies
Geography	E.g., Southern versus Northern England	Wider determinants of health	E.g., Income and work, sick leave and financial support, access to healthy diets and physical activity, education and health literacy
Inclusion health groups	E.g., People experiencing homelessness, people in custodial environments and prisons, migrants, refugees	Access to care	E.g., Access to screening, diagnostic services and treatment services
		Quality and experience of care	E.g., Uptake of screening, experience of breast cancer‐related care, cancer outcomes

*Source*: The King's Fund.^1^

To be pragmatic, the initial search focused on real‐world evidence, including routinely available data sources, such as national cancer registry datasets and key published reports on inequalities by charities, nongovernmental bodies and governmental reviews. Where data were lacking, for example, inclusion of health groups, such as people experiencing homelessness, the briefing also explored grey literature and small‐scale studies.

The briefing aimed to offer practical examples of inequalities rather than to undertake a full systematic review of the available literature. Therefore, there was a risk of subjectivity in preparing the briefing. The technical team documented the key decisions about what to include in the briefing. This was done to assist the quality assurance reviewer in ensuring that an efficient and transparent process was undertaken. Also, in many cases, only single relevant data sources were available due to the lack of data, and overall, there was little scope for selection bias.

At the beginning of the guideline development process, the briefing was provided to various teams at NICE who were involved in breast cancer guidance development. Additionally, an external reference group for breast cancer, consisting of representatives from national bodies and breast cancer services, provided their feedback.

During the initial stages of developing the breast cancer guidance, the NICE committee were given presentations on the health inequalities highlighted in the briefing and an executive summary for quick reference. The committee included various healthcare professionals and lay members, and they were encouraged to consider health inequality issues identified in the briefing when interpreting clinical effectiveness evidence and making recommendations. An online survey was conducted to gather feedback on the briefing's usefulness.

## 3 EVALUATION OF EXPERIENCE

As part of the NICE recent guidance update on diagnosing and managing early and locally advanced breast cancer,^4^ the committee reviewed the evidence and made recommendations on how to reduce arm and shoulder problems after breast cancer surgery or radiotherapy. This followed the standard NICE process and methods related to developing guidelines.^3^

The committee recommended that people receive postoperative supervised support for upper limb exercises in individual face‐to‐face, group or virtual support formats, depending on their circumstances, needs and preferences. The briefing found that people from ethnic minority backgrounds have lower uptake of breast cancer services and are more physically inactive, which may affect their adherence to virtual support sessions. In the rationale for their recommendations, the committee stated that face‐to‐face physiotherapy may be more beneficial for those with complex needs or at higher risk, such as people from minority ethnic family backgrounds.

The committee highlighted in the rationale that there was no effectiveness evidence on outcomes associated with interventions to reduce arm and shoulder problems for different population subgroups, such as people from minority ethnic family backgrounds, disabled people and neurodiverse people. However, they explicitly referred to the EHIA form and the health inequalities briefing, which highlighted, for example, varying levels of engagement with breast cancer services and physical inactivity among different groups. These findings were considered by the committee when making these recommendations, considering their relevance to addressing health inequalities.

The committee also put forward two research areas to tackle health inequalities. First, they suggested further research to identify the most effective and cost‐effective ways of delivering interventions to reduce arm and shoulder problems in individuals who have undergone breast cancer surgery or radiotherapy. The populations of interest were identified as women, men, trans people and nonbinary people, people from minority ethnic family backgrounds, people with learning disabilities or cognitive impairment, physical disabilities, or both, and neurodiverse people. Second, they recommended exploring different intervention formats to determine the adherence and satisfaction levels in the above groups.

The explicit mention of health inequalities issues during committee discussions highlights the usefulness of such health inequalities briefings in informing their decision‐making and keeping health inequalities considerations ongoing during guidance development. The committee feedback was positive and indicated that they were more confident in their discussions about health inequalities. As a result, they were able to make recommendations that addressed health inequalities in a more systematic and transparent way. Also, since there is limited research on health inequalities in general and breast cancer‐specific, an expert review, including stakeholder consultation, was an essential step to ensure the quality and usefulness of this briefing.

It is also worth noting that the approach to addressing health inequalities more systematically in NICE guidance development is still evolving. However, it is anticipated that this briefing will provide valuable information on aspects of health inequalities for all future updates to breast cancer guidance. Other health inequality briefings covering type II diabetes and weight management have been developed. Additional approaches to developing health inequalities briefings are being explored, covering mental health and women's health.

Overall, this approach was well received by the NICE teams involved in the development of breast cancer guidance and the guideline development committee. Other guideline developers could adopt a similar approach to consider health inequalities more transparently and systematically.

Most importantly, the briefing also highlighted that late diagnosis and variation in screening uptake are key drivers of health inequalities among different groups, including deprived women and ethnic minority groups. Although screening decisions fall outside NICE's remit, this information on disparities could provide direction for future national research to improve early diagnosis and screening uptake in groups where screening uptake and outcomes are particularly poor.

## AUTHOR CONTRIBUTIONS

**Eric Slade**: Writing—original draft; methodology; writing—review and editing; supervision; formal analysis; validation. **Kirsty Luckham**: Writing—review and editing; methodology; formal analysis. **Lesley Owen**: Methodology; validation; writing—review and editing; supervision.

## CONFLICT OF INTEREST STATEMENT

The authors declare no conflict of interest.

## ETHICS STATEMENT

Not applicable.

## Data Availability

Data sharing is not applicable to this article as no new data were created or analysed in this study.
